# 'Foot' and 'surgeon': a tale of two definitions

**DOI:** 10.1186/1757-1146-3-30

**Published:** 2010-12-18

**Authors:** Hylton B Menz, Alan M Borthwick, Mike J Potter, Karl B Landorf, Shannon E Munteanu

**Affiliations:** 1 Musculoskeletal Research Centre, Faculty of Health Sciences, La Trobe University, Bundoora, Victoria, Australia; 2 Faculty of Health Sciences, University of Southampton, Southampton, UK; 3 Department of Podiatry, Faculty of Health Sciences, La Trobe University, Bundoora, Victoria, Australia

## Abstract

Recent events in the USA and UK have raised questions about the appropriate definition and application of the terms 'foot' and 'surgeon'. In this editorial, we explore these issues and clarify our use of these terms in the journal.

## Introduction

"When I use a word", Humpty Dumpty said, in rather a scornful tone, "it means just what I choose it to mean - neither more nor less".

Lewis Carroll, *Through the Looking-Glass, and What Alice Found There *(1871)

*Semantics *is defined by the Oxford Dictionary as the branch of linguistics and logic concerned with meaning [1]. However, in a broader sense, the term is also commonly used to describe petty or trivial quibbling over meaning (as in 'semantic arguments'), or to describe the deliberate distortion or twisting of meaning (as in some types of advertising or propaganda). That the very definition of a word that defines the science of meaning is so demonstrative regarding the uses and abuses of language confirms Orwell's assertion that "if thought corrupts language, language can also corrupt thought" [2].

The issue of semantics has recently been highlighted in two separate but related issues of relevance to the foot health professions. Although as the editors of *Journal of Foot and Ankle Research *(*JFAR*) we would prefer not to become entangled in political or legal debates, the very title of our journal in some ways forces us to respond to these issues. The first relates to the very definition of 'foot', and the second relates to the definition of the term 'surgeon'. Both terms, as expected, have been used extensively in the journal since its inception in July 2008, and it is only now that we have felt the need to define them, or at least justify our use of them.

## What connects the foot bone to the leg bone?

The traditional spiritual song *Dem Dry Bones*, thought to have been written by African-American songwriter James Weldon Johnson (1871-1938), has been used for decades to teach basic anatomy to children, and includes the lyric "The foot bone's connected to the leg bone" [3]. Where the ankle fits into this schema could be the topic of an entertaining semantic debate amongst foot health specialists, yet this very question forms the basis of a drawn-out legal case in the USA. The details are freely accessible online [4-7], but what follows is a brief chronology of events related to the case.

In 2000, the Texas State Board of Podiatric Medical Examiners (TSBPME) argued that due to uncertainty among podiatrists, insurance companies and hospitals regarding the scope of practice of podiatry, there was a need to define the word 'foot'. The definition they proposed was:

"The foot is the tibia and fibula in their articulation with the talus, and all bones to the toes, inclusive of all soft tissues (muscles, nerves, vascular structures, tendons, ligaments and any other anatomical structures) that insert into the tibia and fibula in their articulation with the talus and all bones to the toes".

This definition was adopted by the TSBPME in April 2001 [4], despite objections from the Texas Medical Association (TMA) and the Texas Orthopaedic Association (TOA), who claimed that the definition inherently (and impermissibly) expanded the scope of practice of podiatry. The Texan Attorney General concurred, stating that the tibia and fibula are *leg *bones, not foot bones, and as such are beyond the scope of podiatry. The TMA and TOA then filed legal action in November 2002 requesting that the Travis County District Court evaluate the validity of the definition.

In August 2005, the District Court concluded that the TSBPME definition was valid, prompting the TMA and TOA to take the case to the Texas Court of Appeals in March 2008. At this hearing, the court found in favour of the TMA and TOA, and reversed the judgement of the District Court. In their ruling, the Court of Appeals stated that because the definition included parts of the body that were neither part of the foot or the ankle (such as various nerves and blood vessels that traverse the leg and terminate in the foot), the District Court ruling effectively authorised podiatrists to undertake procedures outside of their training, thereby constituting an unauthorised practice of medicine. Interestingly, the ruling did acknowledge that "a compelling argument...might be made as to whether - from a medical standpoint - it is reasonable to allow a practitioner treating the foot to consider and treat other anatomical systems that interact with and affect the foot", although it was specified that a legal case would need to be made to support this [6].

In August 2008, the Texas Podiatric Medical Association (TPMA) and TSBPME appealed the case to the Texas Supreme Court, which was denied in June 2010, and a subsequent request to rehear the case was turned down by the Supreme Court in July 2010 [5]. As a result, the TSBPME definition of the foot is now accepted as being legally invalid. However, legal opinion obtained by the TPMA argues that treatment of the ankle is still within the scope of podiatry practice in Texas, as the definition of the foot specified in the Court of Appeals' decision is "limited to that portion of the body *at or below *the ankle" (emphasis added) [7].

The legal wrangling in this case is clearly driven by scope of practice, professional autonomy, medico-legal and monetary considerations rather than a desire to anatomically locate the talus, and indeed, it is interesting to contemplate how the arguments may have differed if the warring parties were societies of clinical anatomists rather than podiatrists and orthopaedic surgeons. Although the case does not currently have any implications beyond the state of Texas, there is a real possibility that it could set a precedent in other states of the USA, the UK or Australia, or indeed wherever scope of practice conflicts arise between podiatrists and orthopaedic surgeons.

The broader issue raised by this case, however, is that there remains some confusion as to how the foot should be defined. Most dictionaries define the foot by what it is not, i.e. the part of the body *below the ankle *[1]. This is clearly problematic, as the talus could justifiably be considered to be part of both the ankle and the foot. It could also be argued, from a functional anatomy and biomechanical perspective, that no clear distinction between the foot and the ankle can be made, as movement of the ankle joint induces movement in foot joints, and *vice versa *[8]. Even more broadly, there are practical problems with any segmental definition of the foot due to the array of muscular, neural and vascular connections of more proximal structures, although such considerations proved to be the undoing of the somewhat expansive TSBPME definition.

In some respects, *JFAR *has deftly side-stepped the issue of defining the foot by the inclusion of 'ankle' in the title, although we acknowledge that this was merely good fortune rather than careful planning. In considering the Texas case, we have nevertheless reflected on why we decided to include 'ankle' in the journal title. At the time the journal was being developed, several titles were considered which incorporated various permutations of the words 'foot', 'ankle', 'podiatry', 'clinical' and 'research', and we were obviously cognisant of not overlapping with existing journal titles. The journal could have been called *Journal of Foot Research*, but it was assumed (although not explicitly discussed) that because the foot and ankle are interdependent, and that the readership of the journal would be involved in treating ankle as well as foot problems, that *Journal of Foot and Ankle Research *was appropriate. We still believe this to be the case, and therefore feel no need to weigh into a legal, anatomical or semantic debate about where the foot ends and the ankle begins.

## Is a surgeon someone who performs surgery?

Although *JFAR *is the official publication of two podiatry organisations, from its inception we have encouraged submissions from all health professionals, in the hope that the journal would foster greater awareness of, and collaboration between these disciplines [9]. Admittedly, this is a somewhat ambitious goal, and it is certainly too early to evaluate whether it has been achieved. However, we have already received, reviewed and published submissions from several different professions, including orthopaedic surgery, and we are grateful that differences in clinical treatment philosophies and scope of practice do not appear to be a barrier to publishing in the journal. However, it was perhaps inevitable that inter-professional conflicts would eventually arise, which takes us onto the second topic of this editorial related to the problem of definitions.

Earlier this year, we received a manuscript which was subsequently sent for peer review to two referees - in this case, both were podiatrists, one of whom was a podiatric surgeon. As *JFAR *has an open peer review policy, the identity of authors and referees is freely disclosed, and referees are also able to view each other's recommendations. Furthermore, when a manuscript is published, all readers can access the pre-publication history of the manuscript, which includes all referees' comments and the authors' responses. In reviewing the manuscript in question, one of the referees, who works within an orthopaedic centre, queried the use of the term 'podiatric surgeon' and suggested that the authors change this to 'surgical podiatrist'. He justified this request on the grounds that the use of the title 'surgeon' by podiatrists practising foot surgery in the UK is considered controversial, having been the subject of both press and television attention recently [10-12]. In fact, use of the titles 'surgeon' and 'consultant' have been hotly contested for some time [13-17]. Nevertheless, our reviewer's request met with disapproval from the second referee, a podiatric surgeon, who wished to correspond with his co-reviewer on the matter, and asked the editors to provide a contact email address to enable him to do so. Given the open access ethos of the journal, we considered that this was appropriate. Even if we declined this request, a contact email address could have been easily retrieved though an internet search engine.

We were not party to the subsequent correspondence, but presumably the podiatric surgeon would have offered a firm defence of the use of the title 'surgeon' when prefixed by the term 'podiatric'. Indeed, in the light of the recent decision by the Health Professions Council (HPC), the UK's regulatory body for allied health professions (including podiatry) to undertake a public consultation on the annotation of post-registration qualifications, notably for neuropsychology and podiatric surgery, the issue of title assumes even greater significance. In its consultation documentation, the HPC clearly acknowledges that the terms 'consultant podiatric surgeon' and 'podiatric surgeon' have been used within the National Health Service for over 10 years, and that podiatric surgeons are employed in that capacity, and currently use the title [18]. Annotation on the HPC register, however, is also likely to involve legislative protection of a title, and if this is to be the case, presumably the chosen designation will accurately reflect the role whilst avoiding ambiguity. As far as the editors of *JFAR *are concerned, the issue left to consider in this particular case was whether or not to forward (and therefore editorially endorse) the referee's suggested alteration to the authors' use of the title. We decided against doing so, for reasons we will outline shortly. In the end, the paper was not accepted for publication for several reasons unrelated to the use of the title 'podiatric surgeon', but the manuscript did prompt some consideration as to how such an issue would be managed should it arise in the future.

The adoption of definitions and the use of professional titles by journals invariably confer a degree of legitimacy on them, so careful consideration needs to be given when the use of such terms is in dispute. This is particularly true when the journal readership spans two potentially dissenting groups, such as orthopaedic and podiatric surgeons, which are known to contest titles such as 'surgeon' and 'consultant' [14,19]. However, it is not the role of a research journal to takes sides in such debates, which are often conducted in a politico-legal context beyond the scope of the journal's objectives. Indeed, as a journal with an international reach and remit, it is not always helpful, or even desirable, to alter course in response to disputes which emerge in one nation state but not in others. At the same time, it is not always possible to avoid taking a position, and the use of the word 'surgeon' is a clear example of this. After some deliberation, we have taken the view that the term 'podiatric surgeon' is appropriate, based on the simple semantic premise that a surgeon is someone who practices surgery. It could be argued that the suggested alternative, 'surgical podiatrist' is also semantically correct. However, this usage is inconsistent with other applications of the term (e.g. there are *dental surgeons*, not *surgical dentists*) and it is not widely adopted by the organisations that represent these groups, and does not, therefore, adequately perform its descriptive role. The logic behind the term 'surgical podiatrist', as a differentiation from 'podiatric surgeon', is also flawed - the adjective 'surgical' can be defined as 'relating to or used in surgery', which infers that the podiatrist is performing surgery, and the noun for someone who performs surgery is, of course, 'surgeon'. In addition, 'podiatric surgeon' is the phrase used most widely across the Anglophone world to describe podiatrists who are qualified to practice foot surgery. Nor is it evident that the use of the term 'podiatric surgeon' implies, or is intended to imply, that the practitioner is medically qualified as opposed to qualified in podiatry, and therefore designed in some way to obfuscate or deceive, as has been insinuated in some press reports [10-12,17].

Reaching such a decision based primarily on semantics might seem to be taking the easy option. However, we feel that this is the most appropriate approach to take for a journal that strives to be inclusive of all healthcare professionals involved in the management of foot and ankle disorders. Possibly, some of our orthopaedic readers will disagree, and perhaps some of our podiatric colleagues may be dismayed that we have not more emphatically advocated the use of the title on professional or educational grounds. Nevertheless, debates regarding the scope of practice and associated nomenclature of health professions will continue, and the views of the *JFAR *editors are unlikely to significantly influence the outcome. Our job is to get the words right, which is sometimes more difficult than it appears.

## Competing interests

The authors declare that they have no competing interests.

## Authors' contributions

All authors contributed to the writing of the editorial and approved the final submitted version.
